# New “haploid biofilm model” unravels *IRA2* as a novel regulator of *Candida albicans* biofilm formation

**DOI:** 10.1038/srep12433

**Published:** 2015-07-23

**Authors:** Chaminda Jayampath Seneviratne, Guisheng Zeng, Thuyen Truong, Sarah Sze, Wah Wong, Lakshman Samaranayake, Fong Yee Chan, Yan-Ming Wang, Haitao Wang, Jiaxin Gao, Yue Wang

**Affiliations:** 1 Oral Sciences, Faculty of Dentistry, National University of Singapore; 2 Institute of Molecular & Cell Biology, Agency for Science, Technology and Research, Proteos, Singapore; 3 Faculty of Dentistry, The University of Hong Kong, Hong Kong; 4 School of Dentistry, Queensland University, Australia; 5 Department of Biochemistry, Yong Loo Lin School of Medicine, National University of Singapore, Singapore

## Abstract

Clinical isolates of the fungal human pathogen *Candida albicans* are invariably diploid and heterozygous, impeding genetic study. Recent isolation of *C. albicans* haploids opens opportunities to apply technologies unfeasible in diploids. However, doubts remain on whether the haploids, derived from chromosome loss, can represent the diploids. Here, we use *C. albicans* haploids to investigate biofilm, a key virulence attribute. We conducted the first comprehensive characterization of biofilm formation of the haploids in comparison with the diploids. We demonstrate that the haploids form biofilms with essentially the same characteristics as the diploids. Screening a haploid mutant library has uncovered novel GTPase-related genes as biofilm regulators, including *IRA2* that encodes an activator of the Ras GTPase. *IRA2-*deletion mutants develop poorly constructed biofilm in both haploid and diploid *C. albicans*. Our results demonstrate that the haploids are a valid model for *C. albicans* biofilm research and a powerful tool for uncovering novel regulators.

*Candida albicans* is one of the most prevalent fungal pathogens in humans which causes both superficial infections and systemic diseases^1^,^2^. *Candida* species rank as the fourth leading cause of hospital-acquired bloodstream infection and are associated with high morbidity and mortality rates^2^. *C. albicans* forms surface-attached communities known as biofilms, which is a major contributory factor to therapeutic failure due to higher resistance of biofilm mode of growth to antifungal drugs and the host immunity^3^,^4^,^5^.

*C. albicans* possesses an excellent ability to form biofilm communities on medical devices and tissue surfaces, which are extremely difficult to eradicate, giving dire consequences to the patients. Due to its medical importance biofilm mode of growth of *C. albicans* has been extensively studied by various research groups. Last decade, there has been a major drive to develop novel strategies, particularly to combat *Candida* biofilms by academic and pharmaceutical investigators. Therefore, understanding mechanisms governing the biofilm formation and its properties will greatly enhance devising new anti-biofilm therapies against this recalcitrant fungal pathogen. However, a significant difficulty in the study of *C. albicans* biology, including biofilm formation, is the diploid nature of the genome, which hinders the application of forward genetics approaches in identifying novel genes and drug targets. To date, ~4,500 open reading frames in the *C. albicans* genome remain uncharacterized.

Historically, *C. albicans* was regarded as an obligate diploid organism with no haploid state^6^,^7^. Hickman *et al* recently discovered haploid strains of *C. albicans*^8^. This ground-breaking discovery has a profound impact on the current understanding of *C. albicans* biology and pathogenicity. In addition, the availability of *C. albicans* haploid immediately offers high promise to enable, for the first time, applying powerful genetic and molecular approaches previously very difficult or impossible to do in diploid cells^9^. However, as the haploid strains were generated through loss of one set of chromosomes from a highly heterozygous diploid parent, many recessive mutations are unmasked resulting in a genetic background different from their parent. Indeed, the haploid strains show reduced growth rate and diminished virulence compared to their diploid parent, although they do exhibit several key characteristics defining this species, such as the yeast-hyphae transition, the white-opaque switching, and chlamydospore formation^8^. These issues have raised doubts on the suitability of using the haploid strains as research tools, because findings derived from the haploid may not be reproduced in the diploid.

In this work, we performed proof-of-concept experiments. We first comprehensively compared *C. albicans* haploid and diploid strains for their ability to form biofilm, a trait of high importance for life-threatening systemic infection but yet uncharacterized in *C. albicans* haploid. We then constructed a haploid gene-deletion library for genes encoding uncharacterized GTPases and their regulators, and screened the library to look for novel regulators of biofilm formation. We found that the haploid strains are fully capable of forming biofilms, albeit at a slower rate compared to the diploid strains. The haploid biofilm matured and reached a comparable level to the diploid biofilm after three days. The matured haploid biofilm exhibited essentially the same compositional and architectural characteristics as the diploid biofilm. In addition, by screening the haploid mutant library, we identified *IRA2* as a novel positive regulator of biofilm development in both *C. albicans* haploids and diploids. This study, therefore, demonstrates that *C. albicans* haploids can serve as a valid model for the study of biofilm formation and for rapid identification of novel gene functions.

## Results

### *C. albicans* haploid forms biofilm *in vitro*

**Figure 1:**
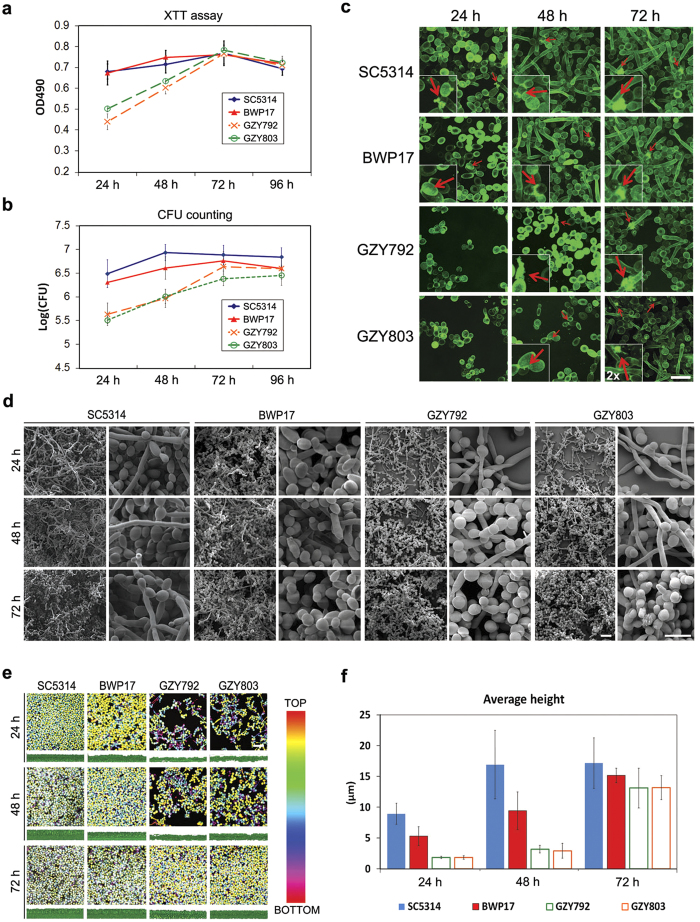
Comparison of biofilms formed by *C. albicans* haploid and diploid *in vitro*. (**a**,**b**) Quantification of the biomass of biofilms formed by diploid strains (SC5314 and BWP17) and haploid strains (GZY792 and GZY803) at different time points with XTT reduction assay (**a**) and CFU counting method (**b**). Cells were cultured in GMM medium (supplemented with required amino acids when necessary) at 30 °C overnight and re-inoculated to allow the development of biofilm at 37 °C. The assays were performed in triplicates, and the means were used to generate the curve with standard error. (**c**) Visualization of extracellular materials (ECM, indicated by arrows) on haploid and diploid biofilms formed at 24 h, 48 h, and 72 h. ConA-Alexa 488 (green) was used to stain the glucose and mannose residues in the fungal cell wall and extended ECM. Bar, 15 μm. (**d**) Visualization of haploid and diploid biofilms formed at 24 h, 48 h, and 72 h with scanning electron microscopy. Bar, 15 μm. (**e**) Confocal imaging of haploid and diploid biofilms formed at 24 h, 48 h, and 72 h. For each time point, the upper panels show the top view (with the depth of biofilm color-coded) and the bottom panels show the side view of the biofilm. (**f**) Comparison of the average heights of haploid and diploid biofilms formed at 24 h, 48 h, and 72 h. Three different sections of each biofilm confocal image were taken for height estimation using BioImage_L to calculate the average height.

To determine whether *C. albicans* haploids can serve as a valid model to study biofilm formation, we carried out the first comprehensive characterization of *C. albicans* haploid biofilms in comparison to their diploid counterparts. We assessed the biofilm forming ability of two haploid strains, GZY792 and GZY803^8^, and two common laboratory diploid strains, SC5314 and BWP17. These strains were cultured according to the published protocol^10^ to allow the development of biofilm. The biofilms formed by each strain were then quantified at 24 h intervals by both the XTT reduction assay (Fig. 1a) and colony forming unit (CFU) counting method (Fig. 1b). The haploid strains were capable of forming mature biofilm communities albeit at a slower rate compared to the diploids (Fig. 1a,b). At 24 h after initial adhesion, haploid cells grew and expanded into microcolonies, and appearance of extracellular materials (ECM) was observed at 48 h. For the diploid strains, ECM was observed at 24 h (Fig. 1c). Scanning electron microscopy (SEM) and confocal laser scanning microscopy (CSLM) imaging revealed that haploid strains demonstrated mature biofilm architecture by 72 h, whereas diploid strains exhibited the same by 48 h (Fig. 1d,e). Mature haploid biofilms and diploid biofilms were structurally similar with spatial arrangements of yeast and hyphal cells embedded in ECM giving the classical three-dimensional appearance of *C. albicans* biofilms. The biofilm comprised of stacks of yeast and hyphal cells with 10–20 μm in height, supported by extracellular matrix, although diploid biofilms contained more hyphal cells by 24 h than haploid biofilms (Fig. 1c–e). We also measured the height of biofilms formed by haploid and diploid strains at different time points. At 24 and 48 h, the average heights of haploid biofilms were substantially less than those of the diploid biofilms. However, the haploid strains could construct biofilms by 72 h with an average height around 13 μm, which was comparable with that of diploid biofilms (Fig. 1f). Taken together, we conclude that the haploid strains are capable of forming biofilms characteristic of the diploid biofilm *in vitro*.

### *C. albicans* haploid maintains its ploidy during biofilm formation

**Figure 2:**
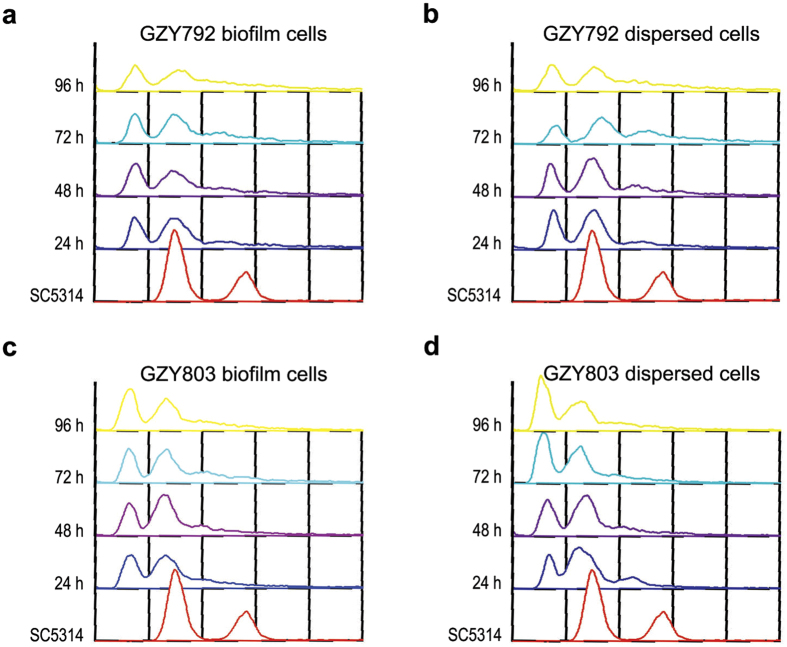
Ploidy examination of haploid biofilms and dispersed cells. Haploid strains GZY792 (**a**,**b**) and GZY803 (**c**,**d**) were cultured at 30 °C overnight and re-inoculated to allow the development of biofilm at 37 °C. Samples were taken from both biofilm (**a**,**c**) and dispersed cells (**b**,**d**) at the time points as indicated for ploidy examination by flow cytometry analysis. SC5314 was used as the standard for diploidy.

*C. albicans* haploids are naturally unstable and tend to auto-diploidize under various culture conditions^8^. Although the haploid tool strains (GZY792 and GZY803) we used for biofilm formation are substantially stable to sustain molecular genetic manipulations^8^,^9^, it remains unclear whether they are also stable during biofilm formation. To address this issue, we took biofilm samples as well as the dispersed cells at different time points during the haploid biofilm development for ploidy assessment by flow cytometry (Fig. 2a–d). Both biofilm and dispersed cells of GZY792 and GZY803 maintained the haploid state during the whole course of biofilm formation (up to 96 h), indicating that the *C. albicans* haploid tool strains are stable in ploidy during the development of biofilms.

### *C. albicans* haploids form biofilm *ex vivo*

**Figure 3:**
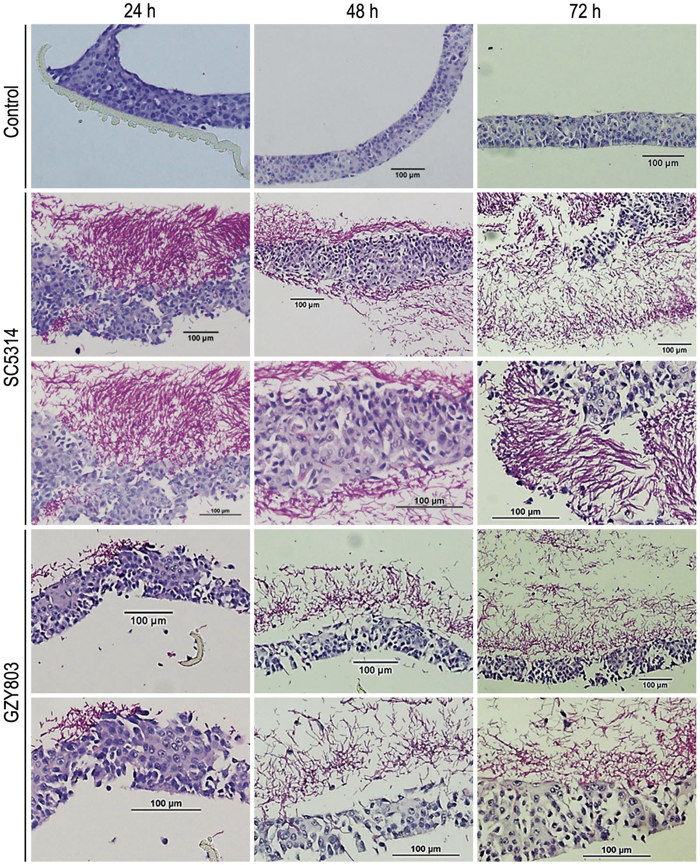
Comparison of biofilms formed by *C. albicans* haploid and diploid on *ex vivo* reconstituted human oral epithelial cells. Reconstituted human oral epithelia were infected with the diploid SC5314 and haploid GZY803, respectively, and incubated at 37 °C with 5% CO_2_. Samples were taken at the time points as indicated and processed for acid-Schiff staining to visualize *C. albicans* biofilm formation and tissue invasion.

In order to ascertain the comparative biofilm formation ability, reconstituted human oral epithelial (RHOE) model was used to examine the *ex vivo* biofilm formation of haploid GZY803 cells in comparison to diploid SC5314 cells (Fig. 3). Consistent with the observations on *in vitro* biofilm formation, the haploid strain was able to form comparable biofilms on RHOE, albeit at a slower rate compared to the diploid strain. The diploid strain formed a thick layer of mature biofilm on the oral epithelial surface by 48 h, whereas the haploid strain formed the same by 72 h. Fungal invasion into the tissues could be detected as early as in 24 h biofilm, which was more obvious in diploid strains. Tissue invasion by GZY803 cells became obvious by 48–72 h. Together, these results further support the validity of using *C. albicans* haploids as a new tool to study biofilm formation.

### Screen of a haploid mutant library for new biofilm regulators

**Figure 4:**
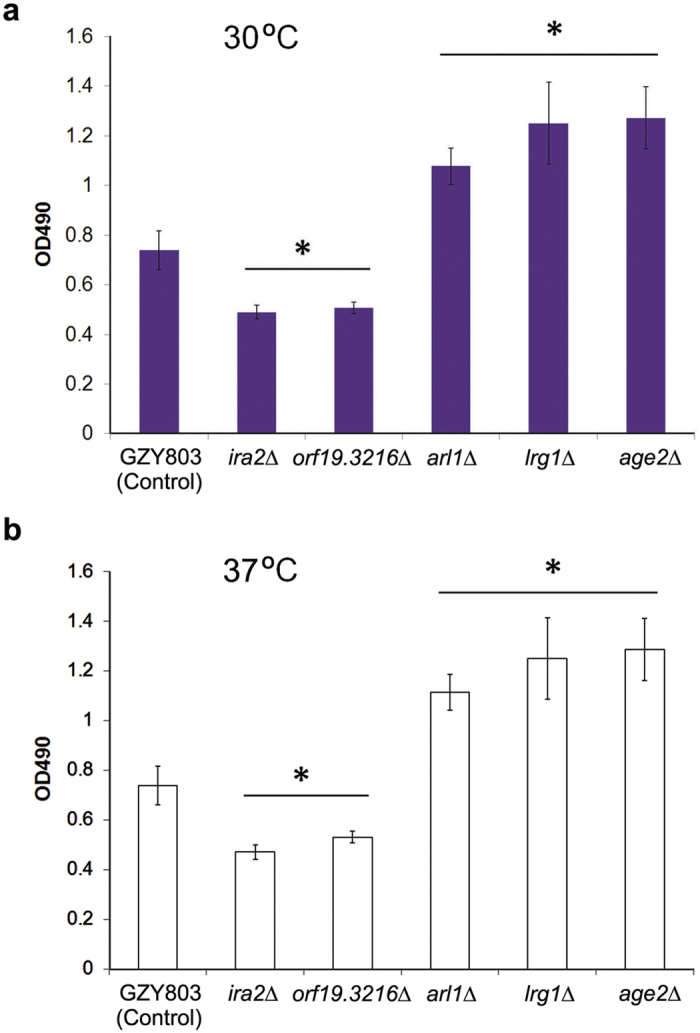
Comparison of biofilms formed *in vitro* by different *C. albicans* haploid mutants with their parental strain GZY803. (**a,b**) Haploid mutants *ira2Δ*, *orf19.3216Δ*, *arl1Δ*, *lrg1Δ*, *age2Δ* and the parental strain GZY803 were cultured at 30 °C overnight and re-inoculated to allow the development of biofilm at either 30 °C (**a**) or 37 °C (**b**) for 72 h. The biomass of biofilm formed by each strain was quantified with XTT reduction assay. Each sample was tested in triplicates and the means were used to generate the bar with standard error. (*): p-value < 0.05.

Next, we utilized this novel haploid *C. albicans* biofilm model to identify novel regulators of biofilm formation. GTPases are modulators of hyphal formation in *C. albicans*^11^,^12^, which could influence biofilm forming ability^13^. However, little is known about the role of GTPases in regulating *C. albicans* biofilm formation. Our group recently established an efficient protocol to rapidly generate specific gene knock out mutants in *C. albicans* haploids^9^, which greatly facilitates the discovery of novel gene functions. Using this new methodology, we constructed from the haploid strain GZY803 a gene-deletion library for 35 putative GTPases and GTPase regulators with unknown functions and screened the mutants for defects in biofilm formation using the newly established haploid biofilm model. Biofilm was allowed to develop in GMM medium supplemented with required amino acids at two different temperatures, 30 °C and 37 °C, to select temperature-independent regulators from the mutant library. Biofilm formation was evaluated by XTT assay at 72 h after initial adhesion, since the parental haploid strain (GZY803) and most diploid strains^5^ were capable of forming mature biofilm by 72 h. Readings taken for biofilms formed by each mutant was normalized to the parent strain GZY803. Foregoing experiments revealed that deletion of *IRA2*, *ARL1*, *LRG1*, *AGE2* or the uncharacterized *ORF19.3216* gene significantly affected the biofilm formation in a temperature-independent manner (Fig. 4). Interestingly, deletion of *IRA2* or *ORF19.3216* had inhibitory effect on biofilm formation while deletion of *ARL1*, *LRG1*, *AGE2* seemed to promote the development of biofilm. Similar results were obtained when biofilm was allowed to develop in RPMI, another medium commonly used for *C. albicans* studies (Fig. S1), suggesting that the observed phenotypes are independent of the culture medium.

### Confirmation of *IRA2* as a biofilm regulator in *C. albicans* haploids

**Figure 5:**
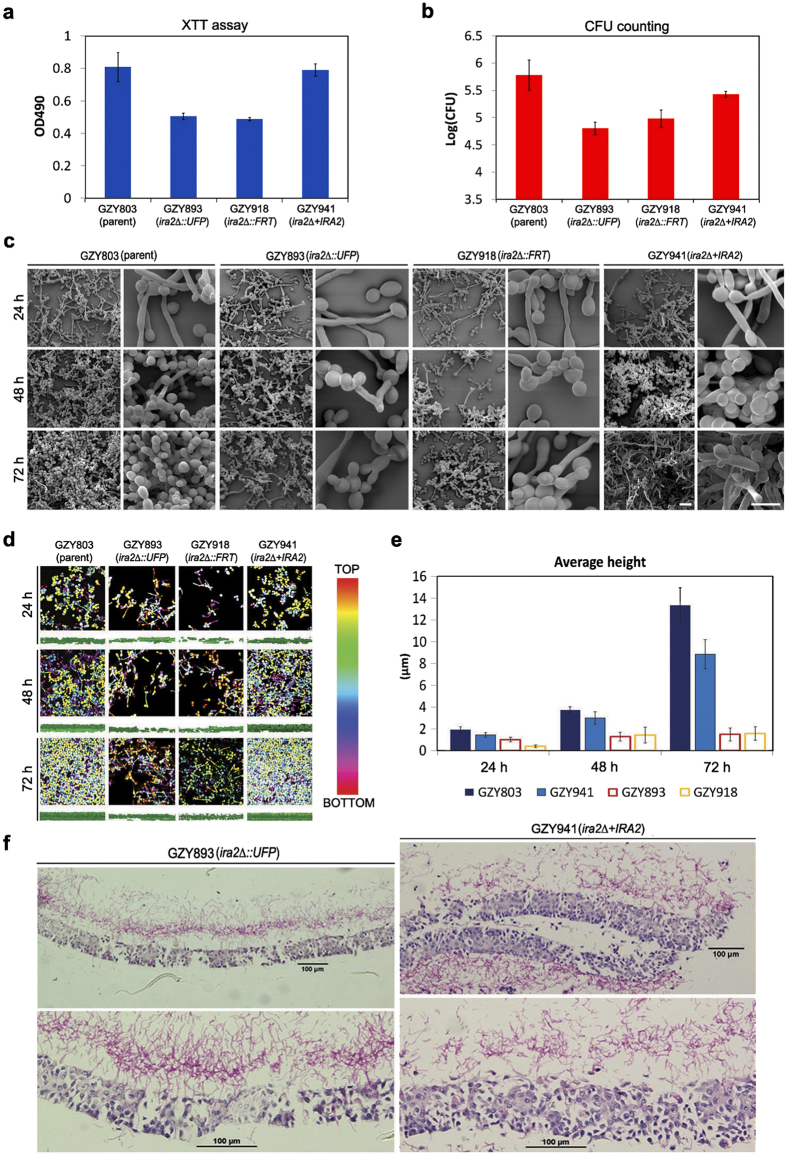
Examination of biofilms formed by haploid *ira2Δ* mutants. (**a,b**) Quantification of the biomass of biofilms formed *in vitro* by haploid *ira2Δ* mutants (GZY893 and GZY918) and the rescued strain (GZY941) with XTT reduction assay (**a**) and CFU counting (**b**). Cells were cultured at 30 °C overnight and re-inoculated to allow the development of biofilm at 37 °C for 72 h. The parental strain GZY803 was used as the control. The assays were performed in triplicates and the means were used to generate the bar with standard error. (**c**) Visualization of biofilms formed by GZY803, GZY893, GZY918 and GZY941 at 24 h, 48 h, and 72 h with scanning electron microscopy. Bar, 15 μm. (**d**) Confocal imaging of biofilms formed by GZY803, GZY893, GZY918 and GZY941 at 24 h, 48 h, and 72 h. For each time point, the upper panels show the top view (with the depth of biofilm color-coded) and the bottom panels show the side view of the biofilm. Bar, 15 μm. (**e**) Comparison of the average heights of biofilms formed by GZY803, GZY893, GZY918 and GZY941 at 24 h, 48 h, and 72 h. Three different sections of each biofilm confocal image were taken for height estimation using BioImage_L to calculate the average height (with standard error). (**f**) Comparison of *ex vivo* biofilms formed by haploid *ira2Δ* mutant and its rescued strain. Reconstituted human oral epithelia was infected with the GZY893 and GZY941, respectively, and incubated at 37 °C for 72 h. Samples were taken and processed for acid-Schiff staining to visualize *C. albicans* biofilm formation and tissue invasion.

Ira2 is a putative GTPase-activating protein of Ras1, an activator of the cAMP/protein kinase A pathway that critically controls the hyphal growth and biofilm formation. However, the function of *C. albicans IRA2* has not been characterized in details, although a previous study had demonstrated that the *ira2Δ/Δ* is not defective in both yeast and hyphal growth^14^. We found that the *ira2Δ* haploid mutant has a similar growth rate like its wild-type parent (GZY803) under biofilm culture conditions (Fig. S2a). In agreement with the previous study^14^, the *ira2Δ* haploid mutant is also not defective in morphogenesis as it exhibited normal yeast cell morphology and produced hyphae indistinguishable from those of the wild-type parent at 37 °C upon serum induction (Fig. S2b). These findings suggest that *IRA2* might have a direct role in regulating biofilm formation instead of acting indirectly via the Ras pathway that regulates the hyphal development. To confirm that *IRA2* is a biofilm regulator in the haploid context, we examined the biofilm formation of both ura^+^ *ira2Δ* (GZY893) and ura^–^ *ira2Δ* (GZY918) strains at 24 h, 48 h, and 72 h relative to their parental strain GZY803 and a rescued strain GZY941. Strikingly, at the mature stage of biofilm formation (72 h), both *ira2∆* strains exhibited about 10-fold reduction in CFU compared to the parental strain, which is consistent with the results of the XTT reduction assay (Fig. 5a,b). SEM and CSLM imaging showed that the *ira2∆* strains formed a meagre biofilm, being thinner and less dense than the biofilm formed by the parental strain (Fig. 5c,d). Estimation of biofilm average height from CSLM was also consistent with the findings (Fig. 5e). When a wild-type copy of *IRA2* was reintroduced into the *ira2∆* mutant, the resulting rescued strain (GZY941) largely restored the biofilm formation features of the haploid strain as demonstrated by XTT, CFU and CSLM results (Fig. 5a–e), suggesting that the biofilm formation defects observed in *ira2∆* mutants are indeed due to the loss of *IRA2* gene function. Consistently, the RHOE model also showed a significantly reduced ability of the *ira2∆* strain to form biofilm in comparison with the rescued strain (Fig. 5f). This comprehensive set of data firmly established an important regulatory role for *IRA2* in regulating biofilm formation in *C. albicans* haploids.

### *IRA2* as a biofilm regulator in *C. albicans* diploids

**Figure 6:**
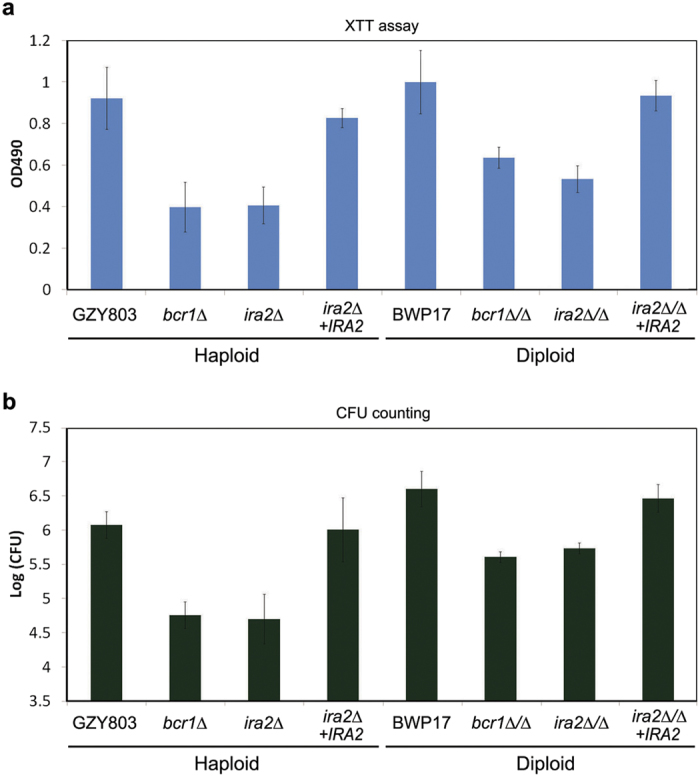
Quantification of biofilms formed by haploid *ira2Δ* and diploid *ira2Δ*/*Δ* mutants. The biomass of biofilms formed *in vitro* by haploid *ira2Δ* (GZY893) and its rescued strain (*ira2Δ+IRA2*, GZY941), as well as diploid *ira2Δ*/*Δ* (GZY921) and its rescued strain (*ira2Δ/Δ+IRA2*, GZY1022) were quantified with XTT reduction assay (**a**) and CFU counting (**b**). GZY803 with *bcr1Δ* (GZY1095), and BWP17 with *bcr1Δ/Δ* (GZY1094), were used as the positive and negative controls for haploid and diploid, respectively. Cells were cultured at 30 °C overnight and re-inoculated to allow the development of biofilm at 37 °C for 72 h.

The above results demonstrated that *IRA2* is a positive biofilm regulator in the haploid background. To examine whether the new biofilm regulators identified from the haploid biofilm model play the same role in *C. albicans* diploids, we constructed an *ira2∆*/*∆* mutant (GZY921) from the diploid strain BWP17 for biofilm formation assays. Previous studies have established *BCR1* as a key biofilm regulator since the *bcr1∆*/*∆* mutant only shows defects in biofilm formation but not in hyphal morphogenesis^15^. Therefore, we included *bcr1∆* haploid (GZY1095) and *bcr1∆*/*∆* diploid (GZY1094) mutants as controls in the experiments. Our XTT and CFU results reflected a consistent observation of the biofilm formation between haploid and diploid mutants (Fig. 6). Both *bcr1*∆ haploid and *bcr1∆*/*∆* diploid strains were defective in forming mature biofilm, thus serving as good negative controls. Similarly, *ira2*∆ haploid and *ira2∆*/*∆* diploid strains both showed a reduction in biofilm formation compared to the haploid GZY803 or diploid BWP17 parental strains, respectively. Again, the defective biofilm formation of *ira2* haploid and diploid mutants could be largely rescued by re-integration of a wild-type *IRA2* gene back to the mutants, further confirming the important role of *IRA2* in biofilm formation. In summary, our results demonstrate that the *C. albicans* haploid strains we developed are powerful tools in the discovery of novel regulators of traits as complex as biofilm formation in this fungal pathogen of humans.

## Discussion

The discovery of *C. albicans* haploids was revolutionary, as this fungus had long been thought to be an obligate diploid^6^,^7^. And, the diploid nature of this organism has impeded application of some powerful genetic approaches routinely used in organisms with a haploid phase in their life cycle^16^, thus greatly hindering progress in *C. albicans* research. There have been doubts on using the haploids to study *C. albicans*, because the haploids carry many unmasked recessive mutations compared to their diploid parent. In this study, we demonstrate the successful use of *C. albicans* haploids to discover new regulators of biofilm formation. We performed the first detailed characterization of *C. albicans* haploid biofilms, and then used the haploids in genetic screens to find novel regulators.

We developed and characterized *C. albicans* haploid biofilms using two models. One was an *in vitro* model using multi-well tissue culture plates, and the other was the *ex vivo* RHOE model which is known to simulate clinical conditions^17^. We observed that the haploid strains followed the same stages as the diploids to develop mature biofilms (Fig. 1), and were able to stably maintain their ploidy during the whole course of biofilm development (Fig. 2). The only difference was that the haploids showed less initial adhesion to the substrate and needed more time to reach a stable mature biofilm community. For most *C. albicans* diploid strains, microcolony formation, sign of early biofilm development stage, is at 3–4 h^5^. Intermediate biofilm development, marked by the emergence of early extracellular material, is at 12 h after initial adhesion. Maturation of diploid biofilm is observed at 38–72 h^5^. The *C. albicans* haploid cells developed the microcolony and intermediate biofilm at 24 h and 48 h, respectively, later than the diploid strains. Nevertheless, the haploid biofilm still reached the mature stage at 72 h. The mature haploid biofilm comprised of metabolically active cells encased in extracellular materials (Fig. 1). This level of complexity in terms of yeast and hyphal architecture of the haploid biofilms was comparable to that of mature diploid biofilms.

RHOE is a well-recognized model for the study of host-microbial interaction and oral candidiasis^17^,^18^. Corroborating the results of the *in vitro* model, the haploid strains formed comparable biofilms to that of diploids on the epithelium at 72 h (Fig. 3). However, we noted a delay in tissue invasion by the haploid cells. The diploid cells had invaded the epithelia by 24 h, whereas the haploid cells showed no clear invasion until 48 h. This indicates that the haploid strain is capable of invading the epithelium in spite of some delay. The delay is probably attributable to the decreased adhesion properties and slower growth of the haploid cells than the diploids^19^. Overall, despite slower development in early stages, the haploid strains eventually formed mature biofilms exhibiting essentially the same cell composition and architecture as the biofilms formed by the diploid strains. Also, we did not detect autodiploidization in the process of haploid biofilm formation (Fig. 2). Thus, our results demonstrate that *C. albicans* haploid strains can be utilized to assess phenotypic features in mature biofilms under appropriate experimental settings.

GTPases are molecular switches involved in the regulation of numerous biological processes. In *C. albicans*, small GTPases such as Cdc42 and Ras1 have been extensively investigated for their roles in regulating hyphal growth and tissue invasion^11^,^12^,^20^,^21^,^22^,^23^. To unravel novel gene functions, we have constructed a haploid gene-deletion library covering mostly genes encoding uncharacterized GTPases and their regulators listed in the *Candida* Genome database (manuscript in preparation). To identify new regulators of biofilm formation in *C. albicans*, we made use of this library to screen for mutants exhibiting altered ability for biofilm formation in this study. Initial screening revealed that deletion of *IRA2*, *LRG1*, *ARL1*, *AGE2* or *ORF19.3216* in the haploid significantly affected biofilm formation at both 30 and 37 °C (Fig. 4). None of these genes have been characterized in *C. albicans*, although some of their homologues have been studied in other fungi such as *Saccharomyces cerevisiae* (Sc). *ScIRA2* encodes a GTPase-activating protein (GAP) that negatively regulates the small GTPase Ras2 which activates the cAMP/protein kinase A (PKA) pathway^24^. *C. albicans* has two Ras proteins, Ras1 and Ras2, that play antagonistic roles in regulating the cAMP/PKA pathway^25^ which governs both hyphal growth and biofilm development^26^,^27^,^28^. Furthermore, *ScIRA2* is a key modulator for cellular response against heat shock, starvation, and dormant cell formation^24^,^29^. The observed involvement of *IRA2* in biofilm formation may help to explain the molecular mechanism underlying the tolerance of *C. albicans* biofilm against extreme conditions. *ScLRG1* encodes a GAP of Rho1 involved in negative regulation of the Pkc1-mediated cell wall integrity signaling pathway^30^, but its role in biofilm has not been examined. *ScARL1* encodes a soluble GTPase having a role in regulation of membrane trafficking^31^, and ScAge2 is an ADP-ribosylation factor GAP effector playing a part in Trans-Golgi-Network transport^31^,^32^. Our observations suggest that membrane trafficking may have an important role in biofilm development^33^. Consistently, the cAMP pathway activates directional membrane trafficking during hyphal development^34^,^35^ which is required for biofilm formation in *C. albicans*^36^.

To confirm that *IRA2* is indeed required for proper biofilm formation, we used two biofilm models and a range of assays, including XTT reduction assay, CFU counting, microscopic imaging and analysis, and confocal computational analysis, to assess biofilm formation of the haploid *ira2Δ* mutant. The results consistently demonstrated defective biofilm formations in the mutant, which could be rescued by reintroducing a copy of wild-type *IRA2* back to the mutant (Fig. 5). When we deleted both copies of *IRA2* in the diploid BWP17 strain, the same defects on biofilm formation were also observed in the diploid *ira2Δ*/*Δ* mutant (Fig. 6), suggesting that the biofilm defects of the haploid *ira2Δ* mutant is directly attributed to *IRA2* gene function rather than a phenotype peculiar to the genetic background of the strain. This comprehensive set of data indicates that *IRA2* plays an important role in the regulation of *C. albicans* biofilm formation.

In addition to *ira2Δ*, four other mutants (*orf19.3216Δ*, *arl1Δ*, *lrg1Δ*, *age2Δ*) have also been found to significantly affect *C. albicans* biofilm formation in the initial screening assays (Fig. 4). The enhanced biofilm formation of *arl1Δ* and *lrg1Δ* mutants might be due to their abnormal morphogenesis during yeast and/or hyphal growth (Fig. S2b), while the enhanced biofilm formation of *age2Δ* at 37 °C is likely a result of its accelerated growth rate (Fig. SFig. S2a). Interestingly, the *orf19.3216Δ* mutant shows a similar growth rate as the wild-type parental strain at both 30 °C and 37 °C, and exhibits normal yeast and hyphal morphologies (Fig. S2), rendering it a good candidate of another previously unknown biofilm regulator. However, the roles of these genes in regulating *C. albicans* biofilm formation remains to be further investigated.

In summary, we have demonstrated that in spite of genetic differences, *C. albicans* haploids can be used as a valid model for the study of complex traits, such as biofilm formation, that characterize *C. albicans* diploids. Importantly, the feasibility to carry out genetic screens using the haploid strains can greatly accelerate discovery of new genes or pathways and speed up identification of novel antifungal drug targets.

## Methods

### Strains, plasmids, and growth conditions

Construction of yeast strains and plasmids used in this study are described in Supplemental Table 1 and 2, respectively. Recombinant DNA manipulations were performed according to standard methods. E.coli XL1 blue (Stratagene) was used as the host strain for recombinant plasmids and cultured in LB broth (0.5% yeast extract, 1% tryptone, and 0.5% NaCl, pH 7.0) supplemented with ampicillin (100 μg/ml). Transformation of *C. albicans* was performed according to the protocol of the Fast Yeast Transformation Kit (G-Biosciences). Targeted gene deletion was performed by transforming the host cells with gene deletion cassette and verified by colony PCR as described^9^. Loop out of *URA3* via FLP-mediated excision followed the previous protocols^37^. Yeast cells were routinely grown at 30 °C in YPD (2% yeast extract, 1% peptone, and 2% glucose), or GMM (glucose minimal medium, 6.79 g/l yeast nitrogen base without amino acids, and 2% glucose) supplemented with appropriate amino acids (uridine 80 μg/ml, arginine 40 μg/ml, and histidine 40 μg/ml) and 5-FOA (1 mg/ml) when necessary. Solid medium plates were prepared by the addition of 2% agar.

### *In vitro* biofilm development

A single colony of each yeast strain was inoculated into GMM medium (supplemented with required amino acids) and grown at 30 ^°^C overnight. The biofilm was developed according to a previous published protocol^10^. In brief, cell suspension was prepared in GMM (supplemented with required amino acids) with the overnight culture at MacFarland standard 0.375 (equivalent to 10^7^ cells/ml). Aliquots of 100 μl of cell suspension were transferred into wells of pre-sterilized flat-bottomed 96-well microtiter plates (Greiner bio-one). The plates were incubated at 30 °C or 37 °C (depending on the nature of the experiment) for 1.5 h with a shaking speed of 80 rpm. After this adhesion phase, non-adhered cells were removed and the adhered cells were replenished with 200 μl of fresh GMM (supplemented with required amino acids). The plates were incubated under the same conditions for up to 96 h, and dispersed cells and biofilm samples were taken every 24 h for evaluation using standard methodologies.

### Biofilm quantification

The biofilm was quantified using 2, 3-bis (2-methoxy-4-nitro-5-sulfophenyl)-5-[(phenylamino) carbonyl]-2H-tetrazolium hydroxide (XTT) reduction assay and colony forming unit (CFU) counting method. After culture medium was aspirated, biofilms were washed with 100 μl of phosphate buffered saline (PBS) once to remove non-adhered cells. XTT assay was performed according to a previously optimized protocol^10^. Briefly, biofilms were incubated with XTT solution (containing 4 μM menadione and 0.2 mg/ml XTT in PBS) at 37 °C and kept in dark for 20 min. The solutions were transferred to a new plate and colorimetric changes were measured at 490 nm using a calibrated spectrophotometer (Multiskan™ GO, Thermo Scientific). In parallel, some biofilms were taken for enumeration of cells by CFU counting method as previously described^38^. Dilution series were prepared from the biofilm suspensions and colonies were counted after incubation at 30 °C for 2 days.

### *Ex vivo* biofilm development

*C. albicans* biofilm formation on *ex vivo* reconstituted human oral epithelium (RHOE) was performed as described previously^39^. Upon arrival, the RHOE was transferred to a 24-multiwell plate (Nunc). The tissues was recovered by incubating at 37 °C overnight in serum-free, chemically defined medium MCDB 153 (Skinethic Laboratory) containing 5 mg/ml insulin, 1.5 mM CaCl_2_, 25 mg/ml gentamicin and 0.4 mg/ml hydrocortison. The tissues was then infected with 200 μl of 10^8^ cells/ml *C. albicans* cells in GMM medium and incubated at 37 °C in 5% CO_2_ for up to 72 h. Samples were harvested at desired time points, washed gently to remove non-adherent *C. albicans* cells and fixed in 4% paraformaldehyde (Sigma) in PBS for 1 h at room temperature. The fixed tissues were then taken and processed for acid-Schiff (PAS) staining to visualize the *C. albicans* biofilm formation and tissue invasion.

### Scanning electron microscopy

Scanning electron microscopy was used to examine the topographic features of the *C. albicans* biofilms formed on coverslips (Thermo Scientific) under similar environmental conditions as described above. The biofilms on coverslips were washed with PBS once and fixed with 2.5% glutaraldehyde (Sigma) overnight at 4 °C. The coverslips were osmicated with 1% osmium tetraoxide for 30 min and dehydrated with a series of ethanol alcohol (70% for 1 h, 95% for 10 min and 100% for 10 min). Coverslips were air dried in a desiccator prior to sputter coating with gold. Afterwards, specimens were mounted on the aluminium stubs and coated with gold in a low-pressure atmosphere with an ion sputter coater (JFC1 100, Jeol). The topographic features of the biofilm were visualized with a scanning electron microscope (Philips XL30CP).

### Confocal laser scanning microscopy

*C. albicans* biofilms were developed on Thermanox (Nunc) 8 well plates under similar environmental conditions as described above. For three dimensional reconstruction of biofilm, live cells in biofilms were stained with SYTO9 and dead cells with propidium iodide (Invitrogen) for 15 min in dark as previously described^38^. The biofilms were observed with an Olympus Fluoview FV1000 TIRF confocal microscope. Z-sections were collected. Images were analysed using Olympus FV10-ASW Viewer and Fiji software^40^. Estimation of the biofilm biovolume was supported by bioImage_L developed by Chávez de^41^.

### Extracellular matrix staining

*C. albicans* biofilms were developed on 8 well chamber glass (Nunc) as described above and samples were taken at different time point. The cells were stained with Concanavalin-Alexa Flour® 488 (Green) (Invitrogen, 100 μg/ml in PBS) by incubating in dark for 20 min as described^5^. The images were taken with an Olympus Fluoview FV1000 TIRF confocal microscope. Z-sections were collected. Images were analysed using Olympus FV10-ASW Viewer and Imaris software (Bitplane).

### Ploidy analysis by flow cytometry

Ploidy of *C. albicans* was analyzed by flow cytometry as previously described^9^. Briefly, adhered and dispersed *C. albicans* cells were collected at different time points during the development of biofilm, washed once with PBS, and fixed with 70% ethanol at 4 °C overnight. Cells were then treated with RNase A (5 mg/ml) at 37 °C for 6–7 h or overnight. After washed once with PBS, cells were further incubated with propidium iodine (5 mg/ml) in dark at 4 °C overnight. The stained samples were sonicated once to separate clustered cells, and subjected to DNA content analysis using BD FACSCalibur. Data acquisition was performed with 10,000 cells for each sample by using the CellQuest Pro software. The acquired data were then analyzed using WinMDI (version 2.8) software. Diploid cells (SC5314) were used as the control to optimize the instrument settings and calculate sample ploidy value.

### Statistical analysis

Statistical analysis was performed using Kruskal-Wallis test, and for any pairwise comparison, Mann-Whitney U test with Bonferroni correction. Analysis was done using SPSS software (Version 16.0, SPSS Inc.). The level of significance was set at P < 0.05.

## Additional Information

**How to cite this article**: Seneviratne, C. J. *et al.* New “haploid biofilm model’’ unravels *IRA2* as a novel regulator of *Candida albicans* biofilm formation. *Sci. Rep.* **5**, 12433; doi: 10.1038/srep12433 (2015).

## Supplementary Material

Supplementary Information
