# Supplementary material for: New “haploid biofilm model” unravels IRA2 as a novel regulator of Candida albicans biofilm formation
Source: Sci Rep. 2015 Jul 23;5:12433. doi: 10.1038/srep12433 (PMC5378891; doi:10.1038/srep12433)
Supplement: Supplementary Information [file srep12433-s1.pdf]

SUPPLEMENTARY INFORMATION

New "haploid biofilm model" unravels *IRA2* as a novel regulator of *Candida albicans* biofilm formation

Chaminda Jayampath Seneviratne[1*+], Guisheng Zeng[2*], Quynh Thuyen Truong[1*], Sarah Sze Wah Wong[3], Lakshman Samaranayake [3,4], Fong Yee Chan[2], Yan-Ming Wang[2], Haitao Wang[2], Jiaxin Gao[2],Yue Wang[2+]

[1]Oral Sciences, Faculty of Dentistry, National University of Singapore, [2]Institute of Cellular & Molecular Biology, Agency for Science, Technology and Research, Proteos, Singapore, [3]Faculty of Dentistry, The University of Hong Kong, Hong Kong, [4]School of Dentistry, Queensland University, Australia

* Equal contribution

+ Corresponding authors

[+]CJ "Jaya" Seneviratne,

Oral Sciences, Faculty of Dentistry,

National University of Singapore

jaya@nus.edu.sg

[+]Yue Wang,

Institute of Cellular & Molecular Biology,

Agency for Science, Technology and Research, Proteos, Singapore

mcbwangy@imcb.a-star.edu.sg

Fig. S1

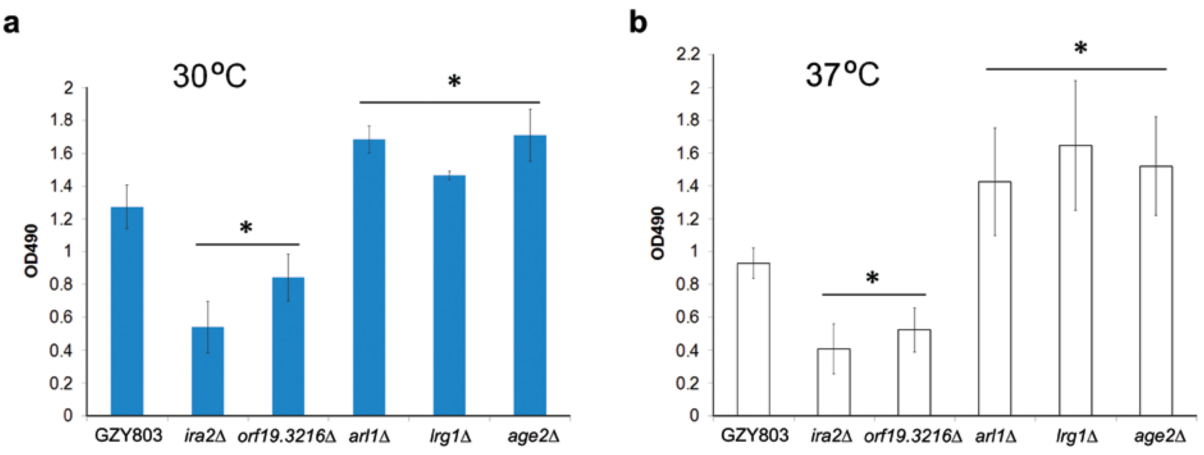

Figure S1. Comparison of biofilms formed in vitro by different *C. albicans* haploid mutants with their parent strain GZY803 in RPMI medium. GZY803 and *ira2Δ*, *orf19.3216Δ*, *arl1Δ*, *lrg1Δ*, *age2Δ* mutants were grown in GMM (supplemented with required amino acids) at 30°C overnight. Each culture was used to prepare an inoculum of $1x10^7$ cells/ml with RPMI medium in a 96-well plate. The plate was incubated at either 30°C **(a)** or 37°C **(b)** with a shaking speed of 80 rpm to allow biofilm development for 72 h. The medium was refreshed every 24 h. The biomass of biofilm formed by each strain was quantified with XTT reduction assay. (*): p-value <0.05.

Fig. S2

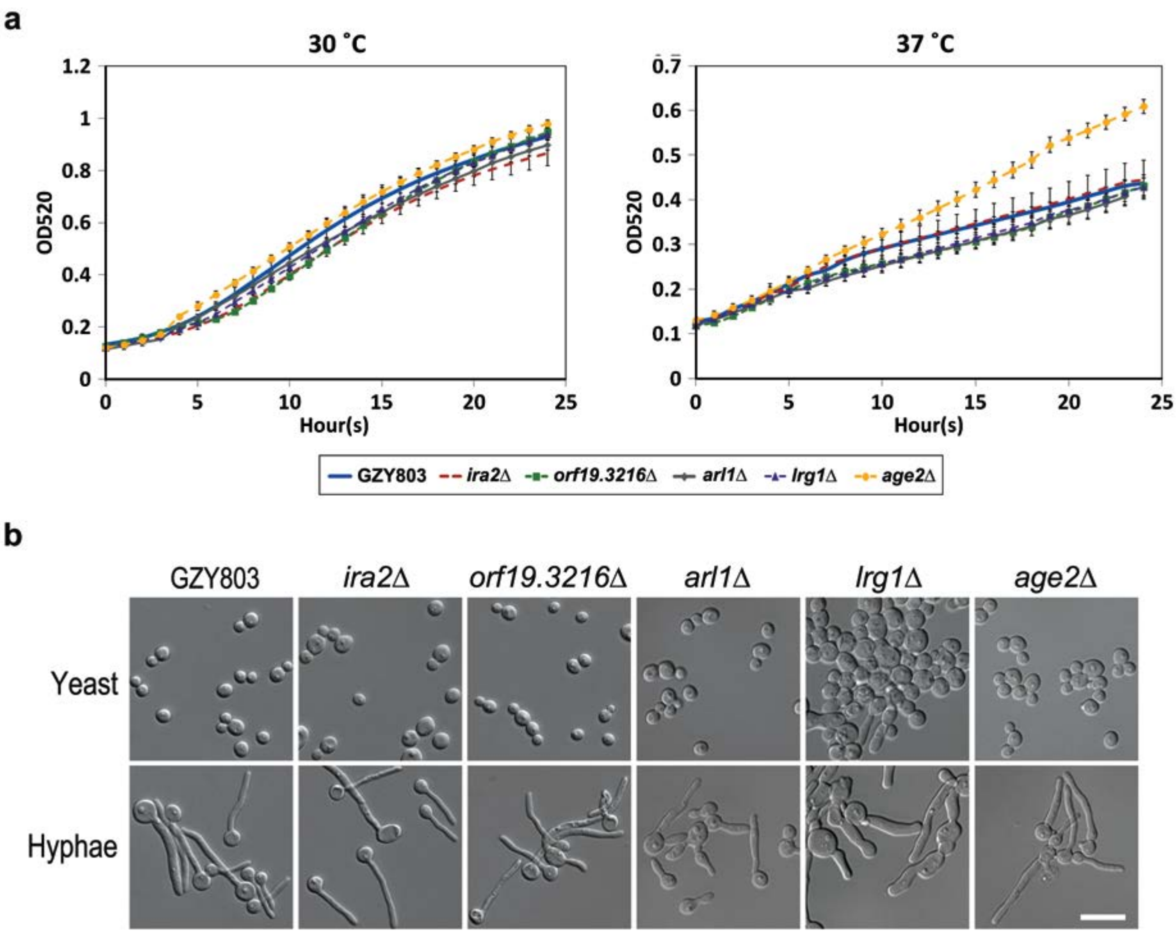

Figure S2: Characterization of the biofilm-regulating haploid mutants. (**a**) Growth rate of the haploid mutants at 30°C and 37°C under biofilm formation condition. GZY803 and *ira2Δ*, *orf19.3216Δ*, *arl1Δ*, *lrg1Δ*, *age2Δ* mutants were grown in GMM (supplemented with required amino acids) at 30°C overnight. Each culture was used to prepare an inoculum with OD520 around 0.1 in a 96-well plate. The plate was incubated at either 30°C or 37°C with a shaking speed of 80 rpm. Optical density at 520 nm for each inoculum was measured in 1 h interval continuously for 24 h. The assay was performed in triplicate and the means were used to generate the growth curve with standard error. **(b)** Morphogenesis of the haploid mutants. GZY803 and the 5 haploid mutants were grown in GMM (supplemented with required amino acids) at 30°C overnight. For yeast growth, the cultures were 1:10 diluted with the same medium and incubated at 30°C for 7-8 h. For hyphal growth, the cultures were 1:10 diluted with the same medium but containing 10% Fetal Bovine Serum and incubated at 37°C for 3 h. Cells were visualized with a Leica DMRXA2 microscope equipped with a CoolSnap HQ2 digital camera and images were taken with the MetaMorph 7.5 software. Bar, 15 µm.

Supplemental Table 1. Yeast strains used in this study

Strain	Genotype	Source strain
SC5314	Wild type diploid	(1)
BWP17	*ura3::imm434/ura3::imm434 his1::hisG/his1::hisG arg4::hisG/arg4::hisG*	SC5314 (2)
GZY792	*MTLα his4*	(3)
GZY803	*MTLα his4 ura3Δ::HIS4*	GZY792 (3)
GZY893	*MTLα his4 ura3Δ::HIS4 ira2Δ::UFP*	GZY803
GZY905	*MTLα his4 ura3Δ::HIS4 orf19.3216Δ::UFP*	GZY803
GZY912	*MTLα his4 ura3Δ::HIS4 arl1Δ::UFP*	GZY803
GZY918	*MTLα his4 ura3Δ::HIS4 ira2Δ::FRT*	GZY893
GZY921	*ura3::imm434/ura3::imm434 his1::hisG/his1::hisG arg4::hisG/arg4::hisG ira2Δ::HIS1/ira2Δ::UFP*	BWP17
GZY923	*ura3::imm434/ura3::imm434 his1::hisG/his1::hisG arg4::hisG/arg4::hisG ira2Δ::HIS1/ira2Δ::FRT*	GZY921
GZY941	*MTLα his4 ura3Δ::HIS4 ira2Δ::FRT IRA2-Myc-URA3*	GZY918
GZY1022	*ura3::imm434/ura3::imm434 his1::hisG/his1::hisG arg4::hisG/arg4::hisG ira2Δ::HIS1/ira2Δ::FRT IRA2-Myc-URA3*	GZY923
GZY1094	*ura3::imm434/ura3::imm434 his1::hisG/his1::hisG arg4::hisG/arg4::hisG bcr1Δ::HIS1/bcr1Δ::UFP*	BWP17
GZY1095	*MTLα his4 ura3Δ::HIS4 bcr1Δ::UFP*	GZY803
WHT11	*MTLα his4 ura3Δ::HIS4 lrg1Δ::UFP*	GZY803
GJX06	*MTLα his4 ura3Δ::HIS4 age2Δ::UFP*	GZY803

1. Gillum, A.M., Tsay, E. Y. Kirsch, D. R. Isolation of the *Candida albicans* gene for orotidine-5'-phosphate decarboxylase by complementation of S. cerevisiae ura3 and E. coli pyrF mutations. *Mol Gen Genet* 198, 179–182 (1984).
2. Wilson, R.B., Davis, D., and Mitchell, A.P. Rapid hypothesis testing with Candida albicans through gene disruption with short homology regions. *J Bacteriol* 181, 1868-1874 (1999).
3. Hickman, M.A., Zeng, G., Forche, A., Hirakawa, M.P., Abbey, D., Harrison, B.D., Wang, Y.M., Su, C.H., Bennett, R.J., Wang, Y., Berman, J. The 'obligate diploid' *Candida albicans* forms mating competent haploids. *Nature*. 7, 55-9 (2013).

Supplemental Table 2. Plasmid constructs used in this study

Construct	Description
CIP10U	*Candida albicans* integration vector with *URA3* as the selection marker; generated by replacing the *RP10* gene in the vector CIp10[1] with 700 bp *GAL1* untranslated region (UTR) at *Pst*I and *Mlu*I sites.
pYGS1089	IRA2Δ::UFP/pBKS; *IRA2* promoter region (~500 bp) and terminator region (~450 bp) were amplified by PCR and cloned into the vector pBKS at *Kpn*I-*Xho*I and *Not*I-*Sac*II sites, respectively, to flank the *URA3* flipper[2] (UFP) located between *Xho*I and *Not*I. The knock-out cassette was released by *Kpn*I and *Sac*II for transformation to generate *ira2Δ::UFP*, which was further used to generate *ira2Δ::FRT* by looping out *URA3* via flippase-mediated excision[2].
pYGS1103	ORF19.3216Δ::UFP/pBKS; *ORF19.3216* promoter region (~500 bp) and terminator region (~450 bp) were amplified by PCR for cloning as pYGS1089. The knock-out cassette was used to generate *orf19.3216Δ::UFP*.
pYGS1108	ARL1Δ::UFP/pBKS; *ARL1* promoter region (~500 bp) and terminator region (~450 bp) were amplified by PCR for cloning as pYGS1089. The knock-out cassette was used to generate *arl1Δ::UFP*.
pYGS1115	IRA2Δ::HIS1/pBKS; *HIS1* was amplified by PCR and cloned into pYGS1089 at *Xho*I-*Not*I to replace UFP. The knock-out cassette was released by *Kpn*I and *Sac*II for transformation to generate *ira2Δ::HIS1*.
pYGS1118	IRA2-Myc-UTR/CIP10U; The *IRA2* gene (-1000 to 7929 bp) was PCR amplified and cloned (between *Kpn*I and *Xho*I) in frame in front of an N-terminal 6x*Myc* epitope (between *Xho*I and *Cla*I), followed by *UTR* (between *Cla*I and *Pst*I), into CIP10U. The plasmid was linearized by *Hpa*I within *IRA2* promoter for integration.
pYGS1250	BCR1Δ::UFP/pBKS; *BCR1* promoter region (~500 bp) and terminator region (~450 bp) were amplified by PCR for cloning as pYGS1089. The knock-out cassette was used to generate *bcr1Δ::UFP*, which was further used to generate *ira2Δ::FRT* by looping out *URA3* via flippase-mediated excision.
pYGS1251	BCR1Δ::HIS1/pBKS; *HIS1* was released from pYGS1115 and cloned into pYGS1089 at *Xho*I-*Not*I to replace UFP. The knock-out cassette was released by *Kpn*I and *Sac*II for transformation to generate *bcr1Δ::HIS1*.
pWHT11	LRG1Δ::UFP/pBKS; *LRG1* promoter region (~740 bp) and terminator region (~700 bp) were amplified by PCR for cloning as pYGS1089. The knock-out cassette was used to generate *lrg1Δ::UFP*.
pGJX06	AGE2Δ::UFP/pBKS; *AGE2* promoter region (~500 bp) and terminator region (~500 bp) were amplified by PCR for cloning as pYGS1089. The knock-out cassette was used to generate *age2Δ::UFP*.

1. Murad, A.M., Lee, P.R., Broadbent, I.D., Barelle, C.J., and Brown, A.J. CIp10, an efficient and convenient integrating vector for Candida albicans. *Yeast* 16, 325-327 (2000).
2. Morschhauser, J., Michel, S. & Staib, P. Sequential gene disruption in Candida albicans by FLP-mediated site-specific recombination. *Mol Microbiol* 32, 547-556 (1999).
